# Influencing factors on ureolytic microbiologically induced calcium carbonate precipitation for biocementation

**DOI:** 10.1007/s11274-022-03499-8

**Published:** 2022-12-28

**Authors:** N. Erdmann, D. Strieth

**Affiliations:** Chair of Bioprocess Engineering, Technical University of Kaiserslautern, Gottlieb-Daimler Str. 49, 67663 Kaiserslautern, Germany

**Keywords:** Biocementation, Biosandstone, Microbiologically induced calcium carbonate precipitation (MICP), Ureolytic activity

## Abstract

Microbiologically induced calcium carbonate precipitation (MICP) is a technique that has received a lot of attention in the field of geotechnology in the last decade. It has the potential to provide a sustainable and ecological alternative to conventional consolidation of minerals, for example by the use of cement. From a variety of microbiological metabolic pathways that can induce calcium carbonate (CaCO_3_) precipitation, ureolysis has been established as the most commonly used method. To better understand the mechanisms of MICP and to develop new processes and optimize existing ones based on this understanding, ureolytic MICP is the subject of intensive research. The interplay of biological and civil engineering aspects shows how interdisciplinary research needs to be to advance the potential of this technology. This paper describes and critically discusses, based on current literature, the key influencing factors involved in the cementation of sand by ureolytic MICP. Due to the complexity of MICP, these factors often influence each other, making it essential for researchers from all disciplines to be aware of these factors and its interactions. Furthermore, this paper discusses the opportunities and challenges for future research in this area to provide impetus for studies that can further advance the understanding of MICP.

## Introduction

**Fig. 1:**
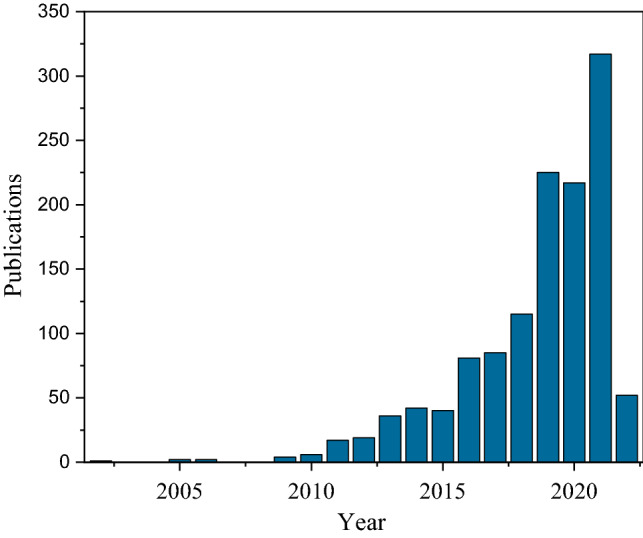
Number of publications per year for the terms “microbi* induced calcium carbonate precipitation”, “microbi* induced calcite precipitation” and “microbi* induced carbonate precipitation”. The data was collected from Scopus in August 2022

With more than 10 km^3^ per year, concrete is the most used building material (Gartner and Macphee 2011). A main component of concrete is cement (CaO), which is produced from limestone (CaCO_3_) at high temperatures (Røyne et al. 2019). During the so-called burning of cement clinker, the temperatures reach about 1450 °C. The energy required for this process is about 2.6% of global energy demand (Miller et al. 2018). Due to resource limitation and a rethinking of ecological issues, there are efforts worldwide to find alternatives to the use of cement. Microbiologically induced calcium carbonate precipitation (MICP) has become an increasingly important research topic in recent years as a possible technology for the consolidation of sand (see Fig. 1).

MICP is a process in which, through the use of microorganisms, carbonate ions (CO_3_^2−^) are formed via various metabolic pathways, which are precipitated as CaCO_3_ in the presence of calcium ions (Ca^2+^) (see Eq. 1). Precipitation occurs when the ion activity product exceeds the solubility constant of CaCO_3_, i.e. the saturation coefficient Ω ≥ 1 (see Eq. 2) (Mondal and Ghosh 2019).

1 $${\text{Ca}}^{2 + } + {\text{CO}}_{3}^{2 - } \leftrightarrow {\text{CaCO}}_{3}$$

2 $${\Omega } = \left[ {{\text{Ca}}^{2 + } } \right]\left[ {{\text{CO}}_{3}^{2 - } } \right]/K_{S,calcium carbonate}$$

If the precipitation occurs in cavities between mineral particles, they can be connected and strengthened. MICP can occur via both autotrophic and heterotrophic metabolic pathways (Castanier et al. 2000). Autotrophic photosynthetic organisms (cyanobacteria, microalgae) and heterotrophic organisms that use the nitrogen cycle (ureolytic organisms, nitrate-reducing organisms) have the greatest potential for MICP (see Table 1). Due to its advantages such as low-cost cultivation in complex media and easy-to-control metabolism, ureolytic MICP is the most widely used mechanism for MICP (Dhami 2013; Muynck et al. 2010a). During this mechanism, one mole of urea is catalytically hydrolyzed intracellularly by urease (EC 3.5.1.5) to one mole of ammonia and one mole of carbamic acid (see Eq. 3) (Anbu et al. 2016). The carbamic acid is then spontaneously hydrolyzed to ammonia and carbonic acid (see Eq. 4). The ammonia diffuses out of the cell and dissociates, forming hydroxide ions. This raises the pH in the area surrounding the cell, which shifts the carbonic acid balance, resulting in the formation of carbonate ions (see Eqs. 5, 6).

3 $${\text{CO}}\left( {{\text{NH}}_{2} } \right)_{2} + {\text{H}}_{2} {\text{O}} \to {\text{NH}}_{2} {\text{COOH}} + {\text{NH}}_{3}$$

4 $${\text{NH}}_{2} {\text{COOH}} + {\text{H}}_{2} {\text{O}} \to {\text{H}}_{2} {\text{CO}}_{3} + {\text{NH}}_{3}$$

5 $$^{ - } 2{\text{NH}}_{3} + 2{\text{H}}_{2} {\text{O}} \leftrightarrow 2{\text{NH}}^{4 + } + 2{\text{OH}}^{ - }$$

6 $$2{\text{OH}}^{ - } + {\text{H}}_{2} {\text{CO}}_{3} \leftrightarrow {\text{CO}}_{3}^{2 - } + 2{\text{H}}_{2} {\text{O}}$$

**Table 1:** Overview of the MICP metabolic pathways that have the greatest potential to consolidate sand

Metabolic pathway	Strains	Pro	Contra	References
Oxygenic photosynthesis	*Gloeocapsa *sp. PCC 73106 (Zhu et al. 2021) *Synechococcus* pevalekii BDHKU 35101 (Sidhu et al. 2022)	Tolerance of most strains to alkaline environment No toxic by-products Cheap media for cultivation	CO_2_ limitation in sand Light dependent Slow growth rates during phototrophic cultivation	Dhami et al. (2014), Zhu et al. (2015)
Ureolysis	*Sporosarcina pasteurii* (Ma et al. 2020; Zhao et al. 2019) *Bacillus sphaericus* (Sharma et al. 2022) *Bacillus cereus*(Oualha et al. 2020) *Bacillus megaterium* (Mukherjee et al. 2022)	Easy to control Cheap media components High calcium carbonate precipitation rates High tolerance of strains to alkaline enviroment	Toxic ammonia as by-product Production of urea coupled to CO_2_ emissions	Hammes and Verstraete (2002), Muynck et al. (2010a), Stocks-Fischer et al. (1999)
Denitrification	*Castellianelle denitrificans* (Jin et al. 2022) *Pseudomonas denitrificans* (Hamdan et al. 2017)	By-products calcium formiate and calcium nitrate are commercial additives for cement Nitrite ions can inhibit corrosion	Usage of carriers for alkaline environments necessary	Erşan et al. (2016)

**Fig. 2:**
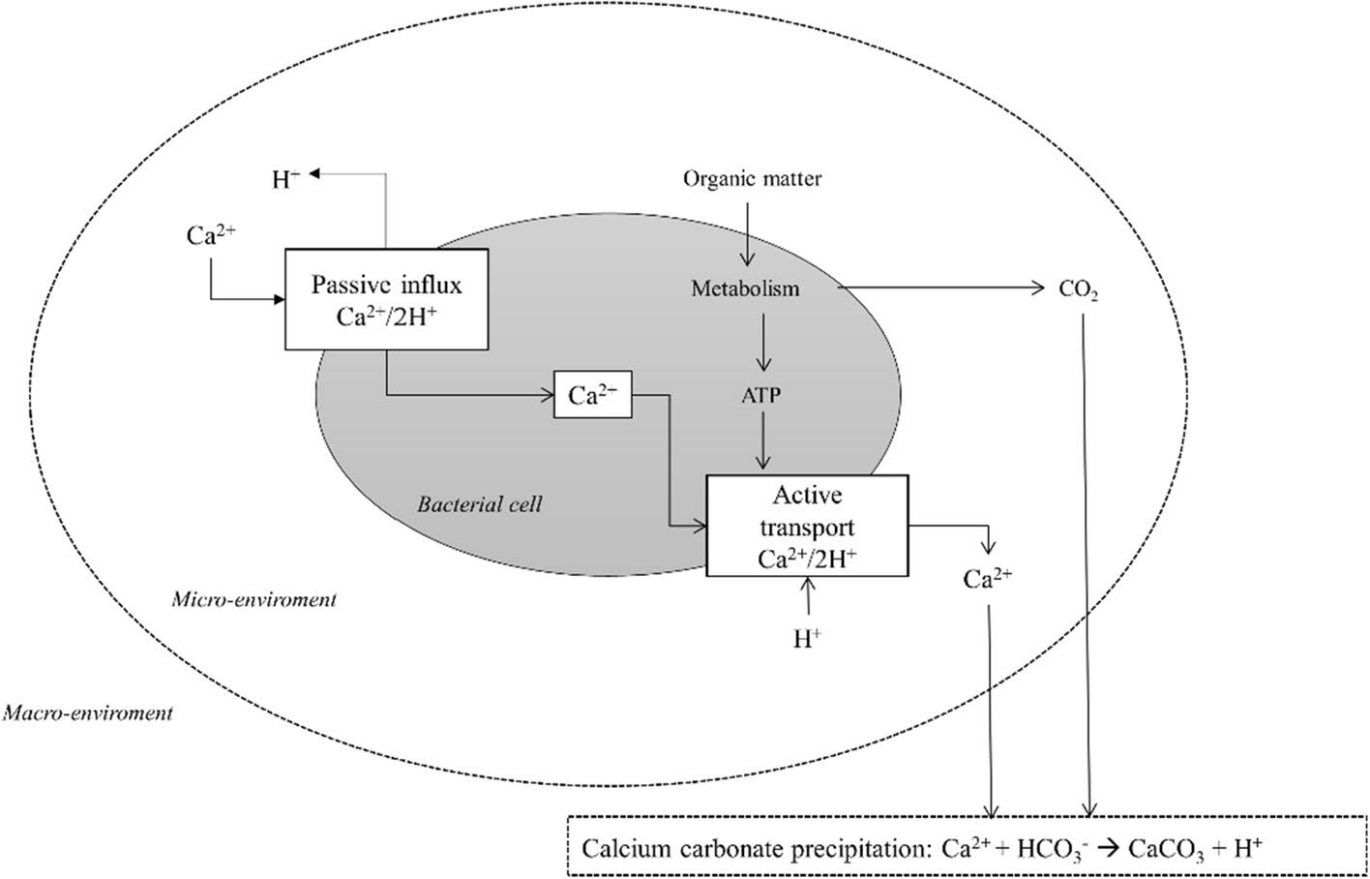
Schematic representation of a proposed calcium metabolic pathway leading to calcium carbonate precipitation modified from Hammes and Verstraete (2002)

Beyond the urea metabolism of the microorganisms, the calcium metabolism also has an influence on the MICP (see Fig. 2). Due to an alkaline environment and a high calcium concentration outside the cell, an electrochemical gradient leads to the passive influence of Ca^2+^ with a simultaneous outflow of protons from the cell.

**Fig. 3:**
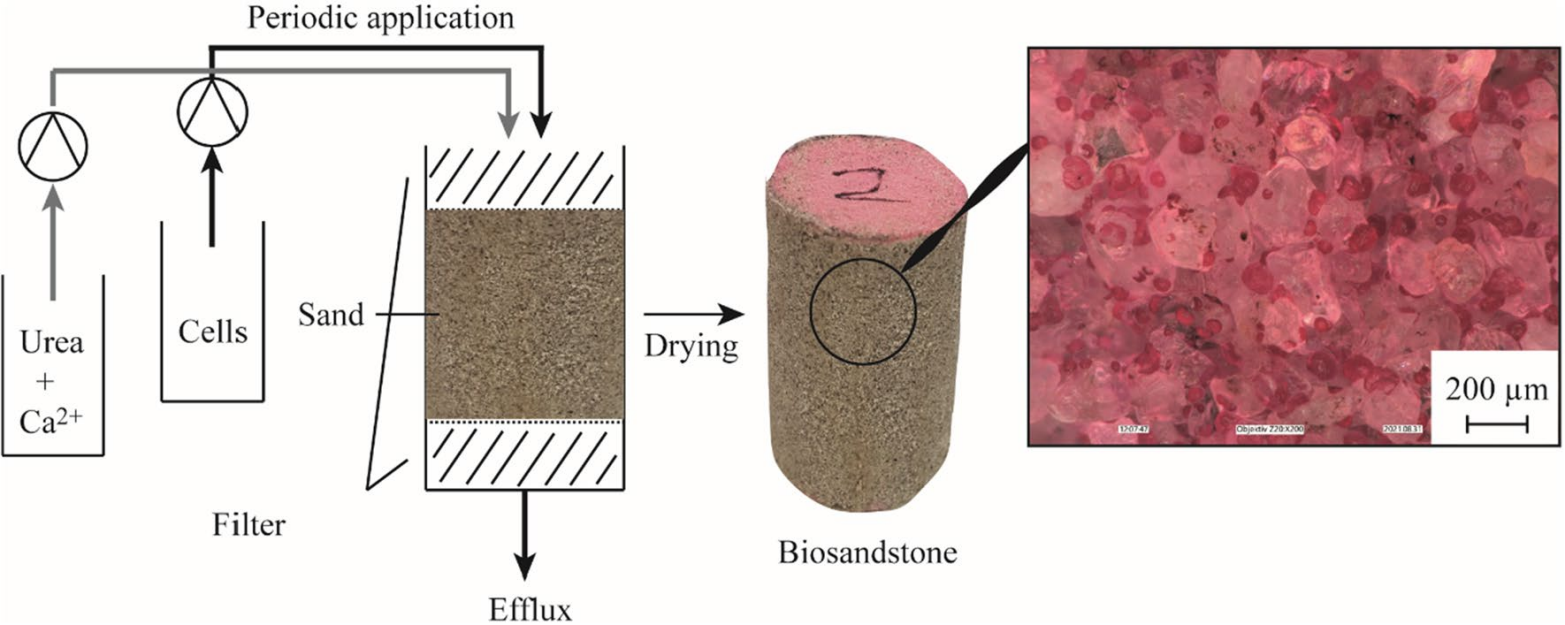
Process for the production of biosandstone modified after Strieth (2022). Right side: Microscopic image of biosandstone after staining calcium carbonate crystals with alizarin red S

Due to the decrease in intracellular proton concentration, the internal pH increases. The high calcium concentration can disrupt several bacterial physiological processes like chemotaxis, cell differentiation and membrane transport as well as signaling processes (Domínguez 2018; Nava et al. 2020). In order to survive under these circumstances, most bacteria export Ca^2+^ by Ca^2+^ exchangers, Ca^2+^/H^+^ or Ca^2+^/Na^+^ antiporters. This process is coupled to an influx of protons and is ATP dependent (Domínguez 2018). As a result of the transport, the concentration of protons in the laminar boundary layer decreases and the concentration of calcium ions increases. A high pH value in combination with high calcium concentration is the optimal basis for CaCO_3_ precipitation. The creation of the advantageous microenvironment results in the formation of crystals on the cell surface, ensuing in the encapsulation of the cell (Muynck et al. 2010a). Although ureolytic MICP is the subject of intensive research, many relationships concerning the interaction of individual influencing parameters during MICP are not fully understood. Various studies have been able to show that MICP has the potential to improve the strength and water permeability of existing construction materials, such as cement mortar and sandstone (Nasser et al. 2022). It also shows the potential to repair cracks in construction materials (Kulkarni et al. 2020; Wu et al. 2019) and to produce materials that could be a more sustainable and ecological alternative to conventional building materials (Mondal and Ghosh 2018, 2019). MICP can also be used to improve the properties of soils for example its liquefaction resistance (Sharma et al. 2022) or resilience against wind erosion (Dagliya et al. 2022). Although some of the influencing factors presented in this study have a general impact of MICP regardless of the application some factors specifically apply to the consolidation of loose sand. Most research in this area uses protocols to consolidate sand in cubic or cylindric moulds. During this process a suspension of ureolytic bacteria and a solution containing a calcium salt and urea are fed into the mould and during curing time CaCO_3_ crystals are formed (see Fig. 3).

**Fig. 4:**
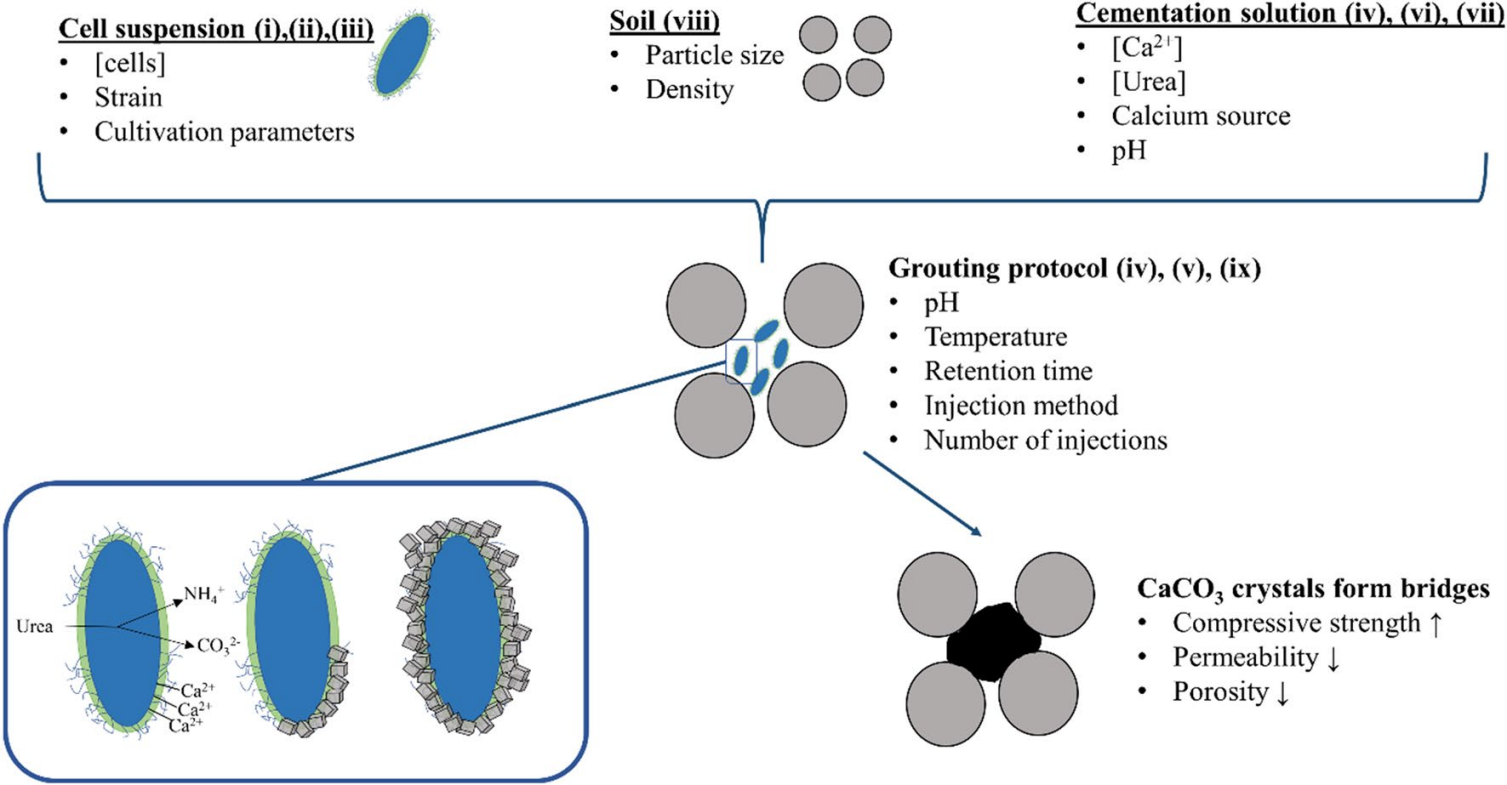
Overview of the influencing parameters during ureolytic MICP for the consolidation of sand

This review summarizes the current research results regarding biological, chemical and physical influencing factors of the ureolytic MICP with regard to the consolidation of sand and highlights the interaction of these parameters. Furthermore, open research gaps as well as limitations and challenges of the technology are discussed. These findings can give impulses for future research projects in the field of ureolytic MICP. Ureolytic MICP is a process that can be influenced by a large number of parameters. In order to improve the process, a basic understanding of these parameters is necessary. A large number of studies have been carried out to identify influencing factors on ureolytic MICP. For calcination of sand, the following influencing factors can be categorized. (i) bacterial strain, (ii) cultivation parameters (iii) biomass and ureolytic activity, (iv) pH, (v) temperature, (vi) concentration of urea and calcium, (vii) calcium source, (viii) sand properties and (ix) grouting protocol (see Fig. 4).

### Bacterial strain

In literature different ureolytic organisms were investigated for their ability to consolidate sand. *Bacillus* and *Sporosarcina* are the most commonly used strains of MICP (Jiang et al. 2019). In general, the choice of organism has an influence on the ureolytic activity which again impacts the morphology of formed crystals. These factors, in turn, have an influence on the result of consolidation of sand. Therefore, the choice of a suitable organism is of great interest for the MICP process. Regarding ureolytic activity, it should be noted that both the choice of organism and the cell concentration influence ureolytic activity during MICP. The influence of biomass concentration will be considered in a separate chapter. Dhami (2013) studied the morphology of formed CaCO_3_ crystals of different isolates from calcereous sand from Anantapur district, India by SEM, EDX and CLSM. They found that depending on the microorganism, there were differences in the size and structure of the CaCO_3_ crystals formed (see Table 2). The authors of the study concluded, based on the results, that the morphology and nature of the CaCO_3_ polymorph are strain-specific.

**Table 2:** Overview of Calcium carbonate morphology and polymorphs of different ureolytic strains. Data collected from Dhami (2013)

Strain	Urease activity in U ml^−1^	Crystal size in µm	Calcium carbonate polymorph	Appearance
*Bacillus megaterium*	690	30–50	Calcite major form Few vaterites	Spherical, oval, rhombohedral, triangular. Smooth and rough surfaces
*Bacillus cereus*	587	15–40	Calcite Major form Very few vaterites	Needle-like, layer-flake structures, irregular square morphology
*Bacillus subtilis*	515	10–50	Calcite Major form Very few vaterites	Spherical, ellipsoidal, very smooth
*Bacillus thuringiensis*	620	2–15	Vaterite major form Few calcite	Round, circular rings, rectangular
*Lysinbacillus fusiformis*	525	2–10	Pure vaterite	Flaky needle like mesh, very rough surface

In a recent study the morphology of CaCO_3_ crystals precipitated from locally isolated strains *Curvibacter *sp. SP-1 and *Arthrobacter *sp. MF-2 were compared (Zhang et al. 2020). For this purpose both strains were cultivated for 50 days in complex medium containing 0.05 M calcium acetate and 0.075 M magnesium acetate. They found an influence of the strain used on the morphology of the crystals. During the cultivation of *Curvibacter *sp. SP-1, mainly dumbbell-shaped crystals were formed within the first days of cultivation, which aggregated into spherical particles during the course of cultivation. *Arthrobacter *sp. MF-2, on the other hand, formed long rod-shaped crystals during the initial phase of cultivation, which also aggregated into spherical particles. However, the surface of spherical particles of *Arthrobacter *sp. MF-2 remained rougher than that of *Curvibacter *sp. SP-1. Calcite was formed by both strains as the main polymorph of CaCO_3_. Crystals formed by *Curvibacter *sp. SP-1 also had an aragonite content of about 20%. Also, amorphous CaCO_3_ formed during the first days of cultivation was converted to crystalline form more slowly in the presence of *Curvibacter *sp. SP-1. These differences in the formation of CaCO_3_ crystals are assumed by Zhang et al. to be due to the different composition of the surface and therefore the surface charge of the strains. How exactly surface proteins or the surface charge influence the polymorphs of CaCO_3_ remains unanswered. Future studies could investigate MICP of genetically modified ureolytic strains which specifically alter the zeta potential or certain knocked out surface proteins. The results could lead to a better understanding of which surface characteristics of microbiological strains have a positive influence on MICP. In follow up studies it will be of interest if tailoring the surface characteristics of these strains can help to achieve certain crystal sizes and therefore different compressive strengths of MICP treated sand. In addition to its influence on the morphology of the CaCO_3_ crystals, the strain used also has an influence on the tolerance of the organism to temperature, salinity and alkalinity during MICP. In order to find an ureolytic organism with a high tolerance for the conditions during MICP and a high yield of CaCO_3_ crystals, a large number of studies in recent years have investigated different ureolytic organisms regarding MICP. For this purpose, not only organisms from strain collections, but also local isolates were investigated in several studies (Ezzat and Ewida 2021; Farajnia et al. 2022; Stabnikov et al. 2013). These studies usually select strains with ureolytic activity comparable to commonly used microorganisms for MICP like *Sporosarcina pasteurii* DSM 33. It appears that halotolerant alkaliphilic ureolytic microorganisms are common in a lot of climate zones with 99% of phylogenic similarity (Stabnikov et al. 2013). Although some of these isolates grow faster than *Sporosarcina pasteurii* (Farajnia et al. 2022), or could achieve 17% higher compressive strengths of biocemented samples than *Sporosarcina pasteurii* DSM 33 there is still no isolate that appears to have a significantly higher potential for MICP than the commonly used strains. However these strains might an impact on local acceptance of the MICP technology. These strains could find more acceptance with local communities and authorities when releasing these strains into the environment while still maintaining similar potential for MICP as *Sporosarcina pasteurii*. Besides the search for organisms that offer advantages over other strains due to their natural environment, few studies have modified strains to investigate the process of MICP. Konstantinou et al. (2021b) developed a method to adjust the urease activity of *Sporosarcina pasteurii*. They inoculated *Sporosarcina pasteurii* in nutrient broth liquid containing no urea and stored this solution at 4 °C. After 3–5 days, fresh nutrient broth liquid was inoculated from this generation and stored at 4 °C. This procedure was repeated until day 42. The authors were able to detect a decrease in urease activity and expression of the Ure-C gene, which encodes for the largest subunit of urease. They compared generations with three urease activities (294, 66 and 19 mmol l^−1^ h^−1^) for consolidation of sand. When using the generation with high urease activity, a highly non-uniform distribution of CaCO_3_ was observed in the samples. Also, clogging of the samples was observed whereby only the upper parts of the samples were consolidated. When strains with lower urease activity were used, an almost homogeneous distribution of CaCO_3_ was observed over the entire sample. The authors emphasize the potential of this method to precisely adjust the ureolytic activity of *Sporosarcina pasteurii* to the application, as they have different requirements for MICP. Furthermore, these results show that ureolytic activity directly impacts the result of MICP which is most likely due to different shapes and sizes of calcium carbonate crystals depending on ureolytic activity. Another way to adapt a microorganism to the needs of MICP application is genetic engineering. However, large-scale application of genetically modified organisms (GMO) outside of controlled laboratories seems unlikely due to local regulatory constraints. However, genetically modified organisms may help to better understand the fundamental mechanisms of MICP. For example did Liang et al. (2018) design 13 plasmids with the urease gene cluster from *Sporosarcina pasteurii* (ATCC 11859) and transformed these plasmids into *Escherichia coli* HB101. They investigated the influence of 4 genes outside the urease gene cluster by deleting these genes resulting in different urease activities and calcium uptake rates for all strains. Therefore, the authors hypothesize that CaCO_3_ precipitation is controlled by more factors of a strain than just its urease activity and that the genes outside the urease gene cluster also have an influence on CaCO_3_ productivity. In a follow-up study Heveran et al. (2019) showed that the morphology and nanomechanical properties of CaCO_3_ can be manipulated by the use of genetic engineering. They compared wild-type *Sporosarcina pasteurii* (ATCC 11859) with two engineered *Escherichia coli* strains HB101/ure-integration and HB101/pBU11 from the study of Liang et al. (2018). *Escherichia coli* HB101/pBU11 was adapted to have urease activity similar to *Sporosarcina pasteurii*, whereas *Escherichia coli* HB101/ure-integration had lower urease activity. During precipitation experiments, only calcite was detected by XRD for all three strains after 7 days. However, for *Sporosarcina pasteurii*, vaterite appeared after 12 h as a transition polymorph. In comparison, for the two *Escherichia coli* strains, no metastable polymorph was observed at any time point. Since *Escherichia coli* HB101, unlike *Sporosarcina pasteurii*, does not form extracellular polymeric substances the authors hypothesized that these stabilized vaterite. They also observed significant differences in the size of CaCO_3_ crystals formed among the strains. While *Sporosarcina pasteurii* crystals had an area of 72 µm^2^, *Escherichia coli* HB101/pBU11 and *Escherichia coli* Hb101/ure-integration reached 16.8 and 340 µm^2^, respectively. The authors concluded that the difference in crystal size is due to the low urease activity, which favors slower crystal growth and thus larger crystals. However, the significant difference in crystal size with similar urease activity of *Sporosarcina pasteurii* and *Escherichia coli* HB101/pBU11 shows that other factors besides urease activity influence MICP. Investigation of the influence of other genes in the region surrounding the urease gene clusters and the composition of extracellular polymeric substances and their influence on crystal formation require more research to advance the understanding of the mechanism of ureolytic MICP. The use of modified ureolytic strains in the recent past has shown that not all mechanisms and influences of MICP can be studied by using wild types such as *Sporosarcina pasteurii*. There is a need in future studies to investigate the influence of individual properties of microorganisms on MICP to better understand the process in detail. Thus, with progressing knowledge of MICP mechanisms, strains can be specifically adapted to meet requirements for the application of MICP. For example, to achieve the highest compressive strength of a construction material, a strain will have to be developed that produces CaCO_3_ in a morphology that creates optimal contact points between individual sand particles to achieve the best consolidation. For the treatment of cracks in existing building materials or for the use of MICP as a natural water barrier, the highest possible decrease in permeability of the treated material would be of importance. Regardless of the application, the use of genetically modified organisms does not appear to be a viable approach for industrial use. Often MICP depends on the application of the organism or a product produced by ureolytic organisms directly in the environment. Large-scale sterilization of artificially produced construction material would be associated with additional energy consumption and would make the application of this technology difficult and less environmentally friendly. The search for suitable wild types and the random mutagenesis of these strains seems to be the more suitable way for an industrial application of MICP.

### Biomass concentration and ureolytic activity

For the application of *Sporosarcina pasteurii* and other ureolytic microorganisms with intracellular urease, the biomass concentration and the urease activity are parameters that cannot influence MICP separately from each other. A higher biomass concentration and thus urease activity, leads to an increased degradation of urea and thus to a faster formation of carbonate ions, which in turn favors a faster CaCO_3_ precipitation (Murugan et al. 2021). However, the use of higher urease activities promotes the formation of smaller CaCO_3_ crystals, which in turn may have a negative effect on the consolidation of sand (Cheng et al. 2017; Wang et al. 2021). There are strong indicators that ureolytic organisms serve the MICP process not only as source of urease but also as initial nucleation sites for crystal growth (Anbu et al. 2016; Ghosh et al. 2019; Muynck et al. 2010b). Positively charged calcium ions in the environment of the microorganisms are attracted by negatively charged carboxy and phosphoryl groups on the cell surface through electrostatic interactions (Seifan et al. 2016). This increases the calcium concentration in the micro environment of the cells. Also, calcium metabolism causes an increase in pH near the cells, which promotes the formation of carbonate ions and thus CaCO_3_ precipitation (see Fig. 2). This is subsequently carried out at this nucleation site in the liquid phase around the cell. Recently it was shown show that during MICP nanometer sized crystals are formed at the cell surface for *Sporosarcina pasteurii* which were confirmed as CaCO_3_ by Energy dispersive X-Ray spectroscopy and X-ray powder diffraction. These results are a further indicator for the role of ureolytic microorganisms as nucleation sites (Ghosh et al. 2019). The influence of these nucleation sites becomes clear by comparing MICP and enzymatically induced calcium carbonate precipitation (EICP). Zhao et al. (2014) were able to show that in aqueous solution for *Sporosarcina pasteurii*, the efficiency of MICP is higher than for EICP regarding the amount of precipitated CaCO_3_. In addition, they found that when comparing the two methods for consolidation of sand, MICP had higher unconfined compressive strength (UCS) in relation to CaCO_3_ content than EICP (see Table 3).

**Table 3:** CaCO_3_ content and unconfined compressive strength (UCS) of samples treated with urease and *Sporosarcina pasteurii*. Modified after Zhao et al. (2014)

Cementation media concentration (1 M urea)	*Sporosarcina pasteurii*	Urease		
	CaCO_3_ content in %	UCS (MPa)	CaCO_3_ content in %	UCS (MPa)
0.25 M Ca	1.90–2.02	0.08–0.18	–	–
0.5 M Ca	7.21–7.88	1.28–1.43	5.12–5.55	0.58–0.62
1.0 M Ca	9.56–11.12	1.60–2.10	6.03–7.02	0.57–0.76
1.5 M Ca	12.14–13.39	2.04–2.13	7.36–8.90	0.79–0.81

The cell concentration and thus the amount of nucleation sites available for formation of new nuclei influence the size of the crystals formed. The formation of new crystals at nucleation sites competes with the growth of already existing crystals. An increase in cell concentration and thus in urease activity leads to a higher number of nucleation sites and thus to smaller crystals, whereas at lower cell concentrations the growth of pre-existing crystals predominates and thus larger crystals occur. In recent studies, Cheng et al. (2017) and Wang et al. (2021) investigated the influence of biomass concentration on crystal size. Cheng et al. found that small CaCO_3_ crystals of about 2–5 µm were formed during calcination of silica sand when a biomass concentration of about OD_600_ 10 was used. At OD_600_ 1, agglomerates of crystals were formed that ranged in size from 20 to 50 µm. Wang et al. also observed an increase in crystal sizes with a decrease in the cell concentrations used. For a cell suspension with OD_600_ = 0.15 they found the largest crystals with a volume of 8000 µm^3^, for a cell suspension of OD_600_ = 1.3 only crystals with a volume of 400 µm^3^. These results are in accordance with the findings of Konstantinou et al. (2021b), Heveran et al. (2019) and Murugan et al. (2021) all of whom found an antiproportional relationship between the urease activity and therefore the OD of an organism and the size of CaCO_3_ crystals formed. Except the study of Konstantinou et al. (2021b) these studies did not differentiate between biomass concentration and ureolytic activity. Since an increase of biomass concentration will lead to an increase of the ureolytic activity and the number of nucleation sites, future research on this topic should try to separate the effects. This could be achieved by mutagenesis of the investigated strain as described by Konstantinou et al. (2021b) or through the utilization of GMO’s. Research with these organisms might help to increase the knowledge especially on the role of the bacteria as nucleation sites which is still not fully understood. This knowledge will help to shape further studies concerning the correlation of morphology and size of CaCO_3_ crystals and mechanical parameters of biocemented sand.

### pH value

The pH of the environment can influence MICP. For example, pH affects the growth of ureolytic microorganisms, urease activity, and CaCO_3_ precipitation. Ureolytic microorganisms in the presence of urea increase the pH in the environment to about 9.25 which is both the optimal pH for growth of *Sporosarcina pasteurii* (Okwadha and Li 2010) and at the same time promotes the formation of carbonate supersaturated conditions in the environment of the cells (Zhao et al. 2019). Furthermore ureolytic activity is pH dependent. Most ureases reach their optimal activity at around pH 8 (Stocks-Fischer et al. 1999). Above pH 8, urease activity decreases. However, since the urease of *Sporosarcina pasteurii* is not extracellular (Whiffin 2004), the pH of the environment plays a minor role for this strain. When using ureolytic microorganisms with extracellular urease, the pH should be considered during MICP to avoid limiting the efficiency of the urease too much. The major influence of pH during MICP is mainly related to CaCO_3_ precipitation itself. CaCO_3_ precipitation is favored by a high concentration of CO_3_^2−^ ions. CO_3_^2−^ ions are in equilibrium with hydrogen carbonate ions and carbonic acid, the so-called lime-carbonic acid equilibrium (see Eqs. 7–9).

7 $${\text{CO}}_{2} + {\text{H}}_{2} {\text{O}} \leftrightarrow {\text{H}}^{ + } + {\text{H}}_{2} {\text{CO}}_{3}$$

8 $${\text{H}}_{2} {\text{CO}}_{3} \leftrightarrow {\text{H}}^{ + } + {\text{HCO}}_{3}^{ - }$$

9 $${\text{HCO}}_{3}^{ - } \leftrightarrow {\text{CO}}_{3}^{2 - } + {\text{H}}^{ + }$$

The balance of this equilibrium, and thus the ratios between the carbonic acid species, is dependent on the pH of the environment. Above pH 10.3 (pH > pK_a_) carbonate ions begins to dominate, favoring CaCO_3_ precipitation. Cheng et al. (2019) investigated the influence of the pH of the calcination solution on the conversion rate of calcium ions to CaCO_3_. They found that the efficiency of MICP remains approximately the same for pH values of 4–8.1, because the degradation of urea increased the pH in the environment, allowing CaCO_3_ precipitation. However, for pH values below 4, there is a rapid decrease in conversion efficiency to 60% (pH 3.5), 10% (pH 3.0) and no CaCO_3_ precipitation could be observed for pH values of 2.5 and lower. In a similar study Lai et al. (2021) investigated the precipitated CaCO_3_ during MICP with *Sporosarcina pasteurii* DSM 33. They also describe a pH value which acts as a deactivation threshold for MICP. For pH values lower than this threshold no CaCO_3_ precipitation occurs. They found that this threshold is dependent on the cell density. Between cell densities of 0.06 × 10^8^ to 10 × 10^8^ cells ml^−1^ the pH threshold rises from pH 2.5 up to pH 4. They also describe that the threshold can be lowered by suspending the bacterial cells into fresh medium before MICP. It is theorized that the cells need adequate nutrients to overcome the severe acidic environment. Which nutrients this might be could not be investigated due to the nature of the complex medium that was used during cultivation of *Sporosarcina pasteurii*. Since initial pH values impacts the initial flocculation time and CaCO_3_ conversion rate (Cheng et al. 2019; Lai et al. 2021). Based on this knowledge Cheng et al. (2019) developed a grouting protocol that utilizes low pH cementation solutions. Further information on this topic will be given in a later chapter. To improve this novel injection method future studies should investigate what nutrients are required to achieve lower pH thresholds. This will help to improve the delay that can be forced until flocculation occurs and thereby give possibilities of injection strategies that were in the past limited due to clogging.

### Temperature

Like other enzymatic reactions MICP is temperature-dependent. The temperature optimum of most ureases lies between 20 and 37 °C (Okwadha and Li 2010) but during MICP the rate of ureolysis is not only dependent on the enzyme activity but also other factors like urea uptake, the release of CO_3_^2−^ and the precipitation kinetics. In the past available kinetic studies on MICP focused on on a single step reaction. They described the kinetic of MICP either through Michaelis–Menten (Lauchnor et al. 2015), zero order (Murugan et al. 2021), or first order kinetics (Lauchnor et al. 2015; Mitchell et al. 2019; Okwadha and Li 2010). While these studies could describe the perceived precipitation rate or urea depletion rate during MICP the kinetic models did not incorporate that the process of MICP has multiple reaction steps staring with urea uptake and ending with the precipitation of CaCO_3_. In a recent study Sridhar et al. (2021) investigated the kinetics of ureolysis of *Sporosarcina pasteurii* DSM 33. They developed a simple structured kinetic model that incorporated urea transport into the cells, hydrolysis of urea, ammonium assimilation and the transport of ammonium out of the cells. It was found urea uptake follows a first order kinetic with a rate constant of 9.8 × 10^–2^ day^−1^ which suggests active transport of urea into cells. They describe the ammonium excretion as free diffusion with a permeability coefficient of 9.2 × 10^3^ m day^−1^. None of these studies did incorporate the influence of temperature into the kinetic model. Investigations of temperature on each single aspect of ureolysis during MICP will be of interest to develop kinetic models that can be used in the future to predict the exact reaction time based on local ambient temperatures In addition to urease activity, temperature also has an effect on the morphology of calcium carbonate crystals. Cheng et al. (2017) were able to show that at a temperature of 50 °C, three times more CaCO_3_ crystals were formed in sand samples during MICP than at 25 °C. However, these had a smaller diameter (2–5 µm) than crystals formed at 25 °C (15–20 µm), resulting in a 60% reduction in the strength of the consolidated sand. Kim et al. (2018) studied the relative CaCO_3_ precipitation for temperatures between 20 and 50 °C. They obtained the maximum precipitated amount of CaCO_3_ for a temperature of 30 °C. As the temperature increased to 50 °C, the amount of precipitated CaCO_3_ decreased by about 50%. While these studies investigated the crystal size directly in the sand bed and the total amount of by CaCO_3_ weight the development of a microfluidic chip (Wang et al. 2019) allowed the observation of crystal growth during different stages of the MICP process in pore spaces. While investigating the CaCO_3_ crystal growth in the temperature range of 4 °C to 50 °C the correlation between temperature and crystal size was found to be dependent on two phenomena. As described earlier the crystal growth is dependent on ureolytic activity. Temperature can therefore impact the crystal morphology indirectly by impacting ureolytic activity. Temperature can also have a direct impact on crystal dissolution behavior following the Ostwald law (Wang et al. 2022). Usually the least dense phase of a crystal is formed first and transformed into the next dense phase. Therefore low temperatures favor the formation of vaterite while high temperatures lead to the formation of the denser and more stable calcite. During this study the average crystal size was the highest for temperatures of 35 °C. The authors of the study state that this is most likely due to higher temperatures reducing cell dennsity and bacterial specific urease activity in their experimental setup and lower temperatures leading to lower chemical transformation efficiency. The potential of this microfluidic chip for the investigation of crystal growth in an environment close to a field of application seem promising. The different findings of temperature influence on the efficiency of MICP might also be dependent on different ureolytic strains. Therefore, it will not only be necessary to find suitable strains depending on ureolytic activity, pH tolerance or the ability to achieve high biomass concentrations during cultivation. Overall temperature is a parameter that is easy to control during MICP and can be altered to achieve certain goals like a faster reaction time or the production of different crystal sizes. However, there is still a need for research on how these effects mutually affect the crystal size and strength of consolidated sand. Furthermore, the contrary results of some of the studies concerning efficiency of the conversion rate of MICP depending on temperature show that it is difficult to investigate certain influencing parameters of MICP independently. For example, while conducting a study on crystal precipitation of MICP not only temperature will impact the results through impact of the observed ureolytic activity and crystal dissolution but also the type of strain that was used and its specific ureolytic activity and mass transport phenomena. Furthermore, the cell density will also impact the results by altering the number of nucleation sites and ureolytic activity during the experiment. It is therefore difficult to compare results of different laboratory setups during these studies. Studies that can truly investigate single influencing factors by disconnecting a factor could help to make these results comparable and increase the overall knowledge of MICP.

### Composition of the calcination solution

Most calcination solutions contain urea, a calcium salt, and are dissolved in either water or a cultivation medium (Amini Kiasari et al. 2019; Qian et al. 2021; Zhao et al. 2019). The urea and calcium concentrations affect the metabolism of ureolytic microorganisms, the efficiency of CaCO_3_ formation, and the morphology of the formed crystals. Since the stoichiometry of CaCO_3_ formation ideally requires an equimolar amount of urea and calcium, most studies are conducted with equimolar concentrations of urea and a calcium salt (Cheng et al. 2017; Ma et al. 2020; Omoregie et al. 2019; Zhao et al. 2019). Optimization of these concentrations has been a central aspect of many studies dealing with MICP. Qabany and Soga (2013) investigated the influence of different equimolar concentrations of urea and calcium in the range between 0.25 and 1 M on MICP. They were able to show that with increasing concentration of the calcination solution, the size of the CaCO_3_ crystals increased from 3–5 µm to up to 35 µm, while the uniformity of the distribution of the crystals and the permeability of the sample decreased. Zhao et al. (2014) treated silica sand with cell suspension of *Sporosarcina pasteurii* and equimolar calcination solutions in the range 0.25 to 1.5 M urea/Ca^2+^. Above a concentration of 0.5 M, they observed a consolidation of the silica sand. The strength of the samples increased with increasing concentration of the calcination solution from 0.6 MPa (0.5 M) to 0.8 MPa (1.5 M) which corresponds to a small increase with a much higher input of resources. However, the authors of the study state that the low concentration of microorganisms with OD_600_ = 0.6 may have limited a higher efficiency at a calcination solution of 1.5 M. In comparison to equimolar calcination solutions, various studies were able to find an improvement in calcination with non-equimolar ratios (see Table 4). For example, Sotoudehfar et al. (2016) found an optimum compressive strength of sand at a ratio of urea to calcium chloride of 3 M to 1.5 M. Muynck et al. (2010a) also investigated the influence of different concentrations of urea and calcium on MICP. Based on their results, they postulated that an optimum of urea and calcium exists for a certain amount of retained cells in sand samples. Up to this optimum, desired effects (consolidation of sand by increasing the concentrations of urea and calcium) outweigh the harmful effects (accumulation of salt and urea in the pores, discoloration of the sand). Han et al. (2021) obtained the best results with an almost equimolar ratio of 0.73 M CaCl_2_ and 0.75 M urea. However, they did not consider the compressive strength of treated sand but the amount of CaCO_3_ that could be precipitated from an aqueous solution. These studies vary in the absolute values of optimum concentrations of the calcination solution but it is apparent that high concentrations above 0.5 M urea and calcium ions are necessary to achieve high compressive strength. This is especially true since compressive strength of biocemented soil correlates with the amount of CaCO_3_ (Rahman et al. 2020) after treatment and the amount of precipitated CaCO_3_ during MICP is directly dependent on the initial concentration of urea and calcium ions and the number of treatment cycles of cell suspension and cementation solution. Compressive strength is not always the most important factor during MICP, for example when environmental impact through high concentrations of the by-product ammonium are a concern. This is true for example for when treating of coastal sand. Ashraf et al. (2021) optimized the cementation solution towards the compressive strength of coastal sand under low concentrations of urea (< 0.1 M) and calcium (< 0.05 M) and achieved compressive strengths up to 154.5 kPa which is described as suitable to strengthen coastal sand into resilient yet habitable ecosystems. While it seems that for MICP it is generally suitable to apply higher concentrations of urea and calcium ions to achieve high strengths there is still more research necessary to understand this process. Especially the correlation between compressive strength, the size of precipitated crystals and homogeneity of samples after treatment are factors that all seem to be influenced by the concentrations of the cementation solution. While these studies were concerned with the compressive strength or the amount of precipitated CaCO_3_, others investigated the influence on the speed of the MICP itself in order to make a statement about the efficiency of the MICP. Okwadha and Li (2010) found the highest rates of ureolysis for MICP at concentrations of 666 mM urea and 250 mM. Onal Okyay and Frigi Rodrigues (2014) reported the highest CaCO_3_ precipitation rate for a calcination solution with a CaCl_2_ concentration of 63 mM and a urea concentration of 700 mM. For all these studies, there are large variations in the CaCO_3_ and urea concentrations described as optimal for MICP. These findings are largely dependent on which parameter is set as the target parameter for MICP. While CaCO_3_ precipitation is fast for low calcium concentrations, since calcium ions inhibit urease activity, MICP stops completely at high concentrations (Whiffin 2004). In this case a large number of treatment cycles must be performed to precipitate a sufficient amount of CaCO_3_, which would not be time and resource efficient. Therefore, in order to make statements about the optimal parameters of the calcination solution, a compromise must always be found between speed and efficiency in terms of the amount of CaCO_3_.

**Table 4:** Optimization approaches of calcination solutions with non-equimolar ratios

Strain	Varied parameters	Target parameter	Optimum (mM Urea/Ca^2+^)	Source
*Sporosarcina pasteurii*	[Urea] [CaCl_2_] Ratio of Cells/calcination solution	Compressive strength	1492 mM Urea 1391 mM CaCl_2_ 7,47 mL cell suspension 7,53 mL cementation solution	Erdmann et al. (2022)
*Sporosarcina pasteurii*	[Urea] [CaCl_2_] NiCl2	Calcium carbonate precipitation rate (h^−1^)	63 mM CaCl_2_ 700 mM Urea 6,9 mM Nickel	Onal Okyay and Frigi Rodrigues (2014)
*Sporosarcina pasteurii*	[Urea] [CaCl_2_]; Reaction time; Flow rate; Cell concentration (OD_600_)	UCS (kPa)	3000 mM Urea 1500 mM CaCl_2_ 20 mL/min OD 4 Curing time 21 days	Sotoudehfar et al. (2016)
*Sporosarcina pasteurii*	[Urea] [CaCl_2_]	Rate of ureolysis (h^−1^)	666 mM Urea 250 mM CaCl_2_	Okwadha and Li (2010)
*Lysinbacillus sphaericus*	[Urea] [CaCl_2_]	Weight gain of sandstone, Permeability	167 mM Urea 227 mM CaCL_2_	Muynck et al. (2010a)
*Sporosarcina pasteurii*	[Urea] [CaCl_2_] [Temperature]	CaCO_3_ precipitated (g l^−1^)	0.73 mol/l CaCl_2_ 0.75 mol/l Urea 45 °C	Han et al. (2021)

Past research could show that the choice of calcium source has an impact on the morphology of calcium carbonate crystals and on mechanical parameters like UCS of treated samples (Achal and Pan 2014; Zhang et al. 2015). In these studies, CaCl_2_ was found to be the optimal calcium source. Since chloride ions have little impact on sandy soil and its low cost, CaCl_2_ is still the preferred calcium source for MICP (Xiang et al. 2022). Samples treated with calcium acetate achieved higher carbonate content than samples treated with calcium chloride or calcium nitrate (Lv et al. 2022). The mechanical strength however is lower which is most likely due more calcite crystals produced with calcium chloride and calcium nitrate compared to the vaterite that was formed during treatment with calcium acetate (Lv et al. 2022). Although calcium acetate seems to be inferior as a calcium source regarding strength of treated samples Xiang et al. (2022) showed that acetate ions react with NH_3_ during MICP which leads to 54.2% and 51.4%, respectively reduced NH_3_ emission in comparison with calcium chloride and calcium nitrate. It should be noted that the utilization of calcium acetate in a seawater environment might be preferable due to less NH_3_ emission, but Peng et al. (2022) found that in seawater environment calcium chloride showed 2% less calcium carbonate production compared to freshwater environment. In comparison calcium acetate showed 7% and calcium nitrate 20% less carbonate production in seawater environment. Nonetheless both environments lead to high strength of the treated coral sand columns. The authors of the study attributed this loss in efficiency to magnesium ions in seawater. These ions promoted the formation of acicular aragonite while in freshwater massive calcite crystals were the main morphology These results show that during the choice of a suitable calcium source the application and environment in which the MICP will be carried out should always be of concern. Although there are still questions regarding the exact mechanisms on the morphology of CaCO_3_ depending on the composition it appears that the composition of the cementation solution is easy to change during MICP and also has one of the biggest impacts on the efficiency of the process. Since the compressive strength of biocemented sand positively correlates with the amount of precipitated CaCO_3_ in the sample (Rahman et al. 2020) there will always be the necessity for multiple cycles of treatment to achieve high CaCO_3_ contents. To keep the number of individual cycles low it will be beneficial to have the highest concentration of urea and calcium in the cementation solution as possible while still maintaining high conversion efficiency of the ureolysis. If ureolytic strains can be found or designed that tolerate high concentrations of calcium during MICP the limitation of the amount of CaCO_3_ precipitated by each treatment cycle would in this case be the solubility of urea and the corresponding calcium salt. This limit can be extended by using higher temperatures of the cementation solution since the solubility of urea and calcium chloride rises with higher temperature.

### Sand properties

While the factors discussed so far have a universal influence on the MICP, different applications of the MICP have additional influences that need to be considered. Regarding the cementation of sand, the particle size and the density of the material impact the result of the MICP. In order to achieve the most homogeneous distribution of CaCO_3_ possible during biocementation, it is necessary for the microorganisms to be able to distribute themselves freely in the matrix before they come into contact with calcination solution. The transport and retention of bacteria in a porous network is dependent on the relative pore throat volume in comparison to the size of individual cells and electrostatic interactions between cells and the particles (Mitchell and Santamarina 2005). Since the pore throat volume is directly dependent on the size of the particles, the size of the particles has an influence on the MICP. Most ureolytic organisms used for MICP have a size in the range of 1–5 µm. If particles smaller than this are used, this can prevent the free distribution of bacteria and thus a homogeneous result of the MICP (Mitchell and Santamarina 2005). Too large particles, on the other hand, have fewer contact points between particles, which does not prevent coarse particles from being consolidated, but increases the effort and amount of resources needed to form a CaCO_3_ layer sufficient to consolidate these particles (Rebeta-Landa 2007). Several studies have investigated the influence of particle size on the efficiency of MICP cementation of sand. Dhami et al. (2016) investigated the consolidation of different sand fractions with grain sizes between 0.1 and 2 mm by *Bacillus megaterium*. For this purpose, they considered the decrease in flow rate through a 76 mm sand bed. For all grain sizes, they observed a decrease in flux rate after 10 days compared to untreated sand. They achieved a larger percent decrease in flow rate for smaller particle sizes compared to larger particle sizes. The largest decrease (88% reduction) occurred for a particle size of 0.5 mm, while only a 66% reduction was observed for particle sizes of 0.2 mm. However, since the initial flow rate at 0.2 mm was 70% lower than at 1.5 mm, because smaller grain sizes have a higher resistance to flow, it is not clear in this study whether these results can also be applied to other parameters that allow statements to be made about the efficiency of the MICP. Cheshomi and Mansouri (2020) compared two silica sands with particle sizes of 0.075–2 mm (S1) and 0.12–0.85 mm (S2). They found that S1 had a higher permeability than S2. As a consequence, S1 showed a homogeneous distribution of CaCO_3_ after MICP, while S2 showed clogging, resulting in an inhomogeneous CaCO_3_ distribution of the samples. The authors concluded that these results were due to the fact that cells were able to move more easily through S1 and thus achieved a better distribution in the sample. In addition to the homogeneity of the distribution, a higher shear strength of 380.6 kPa was also observed in this study compared to S2 (140.2 kPa). Konstantinou et al. (2021a) compared the compressive strength of a fine and a coarse quartz sand with average particle diameters of 0.18 mm and 1.82 mm, respectively, after consolidation by MICP. While for a low degree of calcination (5.5% CaCO_3_) the compressive strengths of the specimens were still close to each other with 500 kPa for fine and 450 kPa for coarse sand, a compressive strength of 2500 kPa was achieved for fine sand and 1600 kPa for coarse sand at a degree of calcination of 10%. Mahawish et al. (2018) investigated the compressive strength of fine (D_50_ = 0.37 mm) and coarse (D_50_ = 9.90 mm) aggregate as well as different blends with 75, 50 and 25% of fine aggregate. Lower compressive strengths were obtained for both pure fine and pure coarse aggregate compared to blends of the aggregates. For a fine aggregate content of 25%, the authors of the study achieved the highest compressive strength of 575 kPa. Further research into the optimum composition of the grain sizes has the potential to make the MICP more efficient without investing in more biomass or urea and calcium ions. Especially interactions between the grain size and density of the soil with the size of precipitated CaCO_3_ crystals during MICP might lead to a better understanding of the overall process and therefore to a reduction in resources used for MICP while maintaining high compressive strength. In addition to particle size, the relative density of the soil also has an effect on the cementation effect of MICP. The relative density D_R_ describes how dense sandy soils are. 0% and 100% indicate the loosest and densest states a sand can assume. Rowshanbakht et al. (2016) and Gao et al. (2019) measured the compressive strength of silica sand after MICP and observed not only a positive correlation between the relative density of the sand and the compressive strength, but also a simultaneous lower CaCO_3_ content of the denser specimens. All of these studies conclude the improved mechanical parameters of the specimens to the fact that compacting the sand brings the particles closer together and thus CaCO_3_ crystals have to bridge shorter distances to consolidate in order to bond the sand particles. Regardless of the choice and particle size of the sand, it therefore seems advantageous to compact the sand before treating the MICP in order to achieve the most efficient increase in mechanical properties. In recent years a transparent microfluidic chip was designed (Wang et al. 2019). The chip allows for a 2D visualization and investigation of processes during MICP. The authors of the study could therefore observe processes like the detachment of bacterial cells from sand grains during flushing with cementation solution and the growth of CaCO_3_ crystals during sequential injections. In future research concerning MICP it will be important to combine these findings of 2D small scale experiments with results obtained in classic column experiments to further develop the knowledge of the multifactorial interactions during MICP.

### Biocementation protocol

**Fig. 5:**
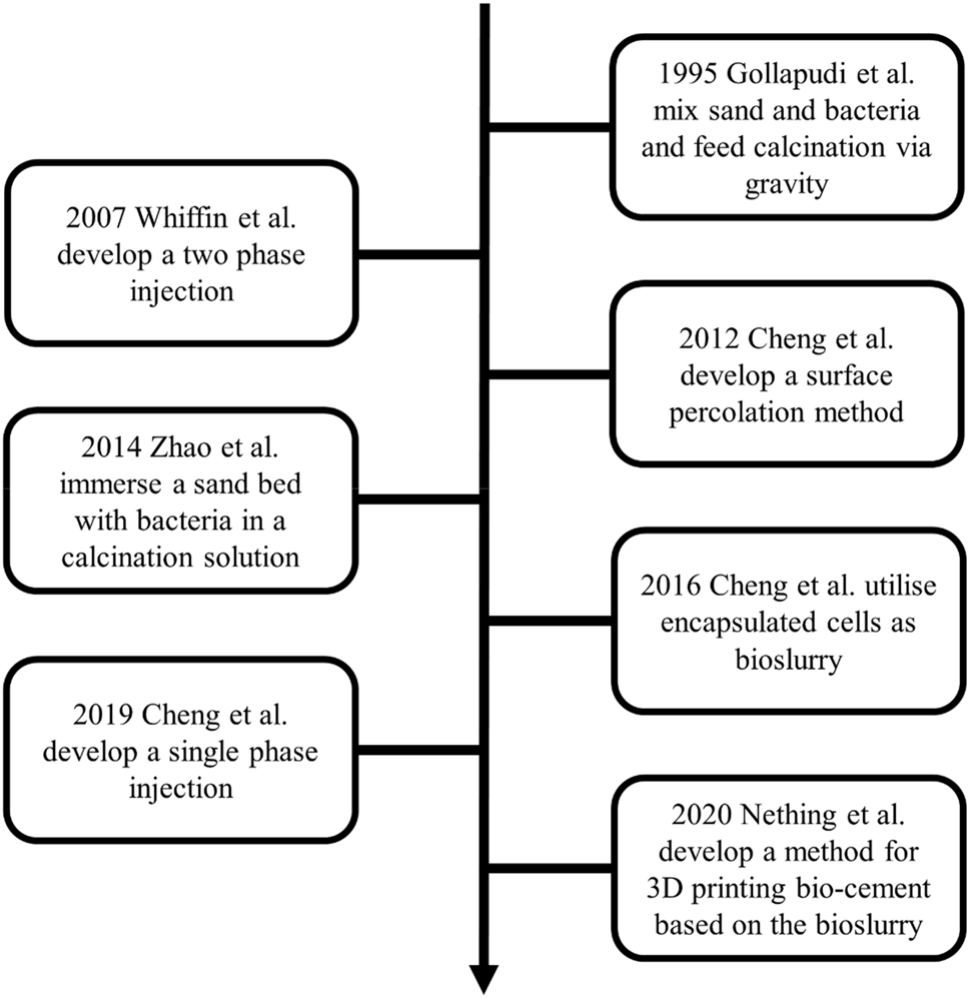
Overview over the development of grouting protocols for MICP

In early studies of MICP, sand was mixed with ureolytic organisms, filled into molds and then treated with calcination solution by gravity feed, or cell suspension and calcination solution were mixed and immediately applied to a sand bed under pressure. (Stocks-Fischer et al. 1999; Whiffin 2004). However, these methods often lead to a clogging of the sample at the entry point of the calcination solution due to the decrease in permeability, which resulted in an uneven consolidation of the samples (Stocks-Fischer et al. 1999; Whiffin et al. 2007). Since then various methods have been developed over the years to perform MICP more efficiently (see Fig. 5).

In 2007 Whiffin et al. (2007) developed a 2-phase injection protocol. Phase 1 is the placement step in which the cell suspension is applied to the sand and fixed. For this purpose, the cell suspension is applied until the mould is completely filled and bacterial cells are observed at the exit point. Then, a pore volume of 50 mM calcium chloride solution is applied to increase the ionic strength and thus increase the adsorption of cells on the surface of sand particles. Immediately after the placement step, a calcination solution is applied under uniform flow. Over the length of a 5 m column of sand, they were able to demonstrate the formation of CaCO_3_ over the full length using this method without causing a complete clogging during the process. However, complete homogeneity was not achieved in terms of compressive strength and CaCO_3_ content of individual sections. With increasing distance from the injection point, the CaCO_3_ content decreased from 85 kg m^−3^ sand to about 3 kg m^−3^ sand. From a distance of 2.5 m from the injection point, they could not detect any increase in the compressive strength of the sand in contrast to untreated samples. Cheng and Cord-Ruwisch (2012) developed a simple surface percolation method for MICP consisting of four steps. (i) percolation of the cell suspension (50% water retention capacity), (ii) percolation of a fixation solution (50% water retention capacity), (iii) incubation for 12 h to allow diffusion processes, and (iv) percolation of the calcination solution (100% water retention capacity). Using this method, they were able to achieve nearly uniform consolidation over a length of 1 m for one column. Cheng and Cord-Ruwisch (2012) combined the percolation method with the two-phase injection of Whiffin et al. (2007) and were able to achieve higher localized strength over the entire length of the specimen with the percolation method than with the two-phase injection. However, inhomogeneities in CaCO_3_ content along the length of the specimen were also observed for this method. While consolidation of sand could be achieved with these methods and the problem of complete clogging could be eliminated, effluent reduces the number of bacteria in the sample and the problem of unevenly precipitated CaCO_3_ remains. Zhao et al. (2014) developed a novel method by using full contact flexible molds made of geotextile, a polypropylene, staple fiber and needle punched nonwoven material. Sand is mixed with ureolytic bacteria for this method and filled into the molds. The moulds are then completely immersed in a reactor filled with calcination solution consisting of CaCl_2_, NH_4_Cl, NaHCO_3_ and Nutrient Broth (Mortensen et al. 2011). Through the pores in the mold, the calcination solution can soak into the specimens and the specimens are consolidated by ureolytic MICP. Using this method, the authors were able to achieve a compressive strength of 2 MPa with nearly homogeneous CaCO_3_ concentration in the specimens. However, a disadvantage of this method is that no further cells can be introduced into the moulds during curing and the amount of precipitated CaCO_3_ is thus limited by the amount of cells applied, which encapsulate and die over time (Muynck et al. 2010b). Wen et al. (2020) were able to solve this problem by further developing the method. They took the samples after immersion, dried them for 24 h at 105 °C and then treated them again with cell suspension and calcination solution in a batch reactor. This allowed them to increase the compressive strength of the samples from 2.2 to 6.4 MPa. Cheng and Shahin (2016) were also able to achieve a uniform distribution of microorganisms by using a so-called bioslurry. To prepare the bioslurry, a cell suspension of *Bacillus *sp. DSM 23526 was mixed with a solution of urea and calcium chloride. The cells then precipitated CaCO_3_ and were encapsulated. After sedimentation of the crystals, they were separated from the supernatant to harvest the bioslurry. For biocementation, sand was mixed with bioslurry at a ratio of 10:1 and placed in a PVC column. The column was flushed up to eight times with a calcination solution, resulting in a UCS of 1.1 MPa. Over a column length of 275 mm, a uniform CaCO_3_ content of 0.055 g CaCO_3_ g^−1^ sand was achieved. However, this sample also has the disadvantage that the amount of cells is limited during MICP and thus no further CaCO_3_ precipitation takes place after encapsulation of the cells. A possible approach to achieve a homogeneous distribution as well as to provide a sufficient amount of cells in a sample was developed by Cheng et al. (2019). This approach takes advantage of the dependence of MICP on pH as described earlier. By lowering the calcination solution to pH 4, a lag phase could be generated before the ureolytic activity of microorganisms causes the pH to rise and CaCO_3_ precipitation to occur. This allows calcination solution and cell suspension to be mixed and then immediatly be applied to a column of sand without being limited by clogging. Using this method, Cheng et al. (2019) were able to consolidate silica sand to a compressive strength of 2.5 MPa using *Sporosarcina pasteurii* and achieved a nearly homogeneous distribution in a 360 mm column. An advantage of this method is that the atmospheric ammonium released was reduced by 90%. However, this method has a limitation in application at low cell concentrations and ureolytic activities, as these were not able to raise the pH of the environment to a level that allowed MICP to occur. However, this study could not answer whether the results could be transferred to other ureolytic organisms. It may be possible to extend the lag phase by using organisms that tolerate lower pH values, which could further increase the efficiency of the method. Another approach to improve the homogeneity of the samples could be the approach of 3D printing. Nething et al. (2020) combined the method of bioslurry with the method of immersion in their study on the feasibility of 3D printing using MICP. For this approach, they lyophilized the bioslurry to obtain a ureolytically active powder. A sand bed was now printed over a powder print. In the areas that were to be consolidated using MICP, the sand was mixed with the bioslurry powder via a screw thread. The entire sand bed was then immersed in a calcination solution. Via this method, the authors were able to print geometrically stable 3D structures. This overview of grouting protocols shows that there is a steady evolution in MICP methods. This is mainly driven by the fact that the protocols often lead to non-uniform distributions of CaCO_3_ in the samples due to blocking or washing out of cells. Especially regarding the application as sustainable building materials, suitable protocols have to be found that can reproduce the materials with high precision to not allow weak spots due to inhomogeneous samples. The development of new strains and a deeper understanding of the mechanism of MICP may allow in the future to develop new grouting protocols precisely adapted to a specific use case of MICP. The issue of clogging and homogeneity of samples in a lot of cases is still a problem that needs to be addressed in future research. To improve these protocols, it will be necessary to understand the process of MICP in detail. Recent research on the topic of ureolytic activity can be incorporated to find the optimal curing time between treatment cycles which is often still overestimated to give the process enough time to fully degrade urea. Especially the understanding of microscopic processes during MICP through microfluidic chips could lead to the development of customized parameters. While in the past it was difficult to see and conduct research on the growth of CaCO_3_ crystals in the sand matrix it will be possible to get visualization of this process. This will help to develop new protocols not only based on parameters of treated samples but also through understanding of the behavior of the microorganisms, especially their attachment to particles and flow through the pore spaces during injections, and the interactions of CaCO_3_ crystals with sand grains.

## Limitations and challenges

Although there are promising results in laboratory and even field scale application of biocementation by MICP there are some issues that need to be addressed for a widespread use and acceptance of biocement in industrial scale. During ureolytic MICP ammonium is produced in high amounts as a by-product (see Eqs. 3–6). Ammonium can have a serious negative impact on the environment and human health. Although this might not be a problem for laboratory scale production of biosandstone a widespread application of MICP would lead to significant waste streams containing ammonium. A promising solution for this problem lies in the utilization of the mineral struvite (NH_4_MgPO_4_⋅6H_2_O). Yu et al. (2016, 2018) demonstrated that struvite precipitation can similar to CaCO_3_ be induced by ureolytic microorganisms. For the biomineralization they used *Sporosarcina pasteurii* and a solution of MgCl_2_, K_2_HPO_4_⋅3H_2_O and urea. The precipitated struvite acts similar to CaCO_3_ as a binder between particles. Instead of precipitating struvite directly in the sand Gowthaman et al. (2022) treated the effluent of MICP experiments with Mg^2+^ and PO_4_^3−^ and could achieve a reduction of around 90% of ammonium from the effluent through struvite precipitation. Other alternatives for the treatment of ammonium rich efflux could lie in treatment of the efflux by anaerobic ammonium oxidation (Mao et al. 2017) or the usage of zeolites during MICP to reduce the ammonia leakage (Su et al. 2022). In any case the utilization of ammonium will be necessary for widespread application of MICP. The treatment of efflux during production of construction materials by MICP will generally be easier to handle than in other biomineralization applications like soil remediation where the ammonium will be directly released in the environment. Additionally to the challenge of treating the toxic by-product during MICP the environmental impact needs to kept low for a technology that is supposed to be an ecological alternative to conventional cement. In lab scale research on MICP for the cultivation of ureolytic microorganisms mainly lab grade chemicals like yeast extract or nutrient broth are used (Omoregie et al. 2019). Since these chemicals are expensive a transition to industrial scale application of MICP will only be possible if suitable alternatives to these chemicals are found. Although expensive these cultivations usually lead to low or medium biomass concentrations. Lapierre et al. (2020) studied the nutrient requirements of *Sporosarcina pasteurii* in a defined medium in detail and published a supplemented complex medium that lead to an increase of cell density of about 400% by only increasing the price of the medium by about 4.3%. Another approach to obtain biomass of *Sporosarcina pasteurii* is upcycling of waste streams for example diary and brewery waste (Cuzman et al. 2015; Kahani et al. 2020). By combining the knowledge of suitable waste streams and nutrient requirements of the strains, protocols for cultivation of ureolytic bacteria can be improved which will help MICP to become a truly environmentally friendly alternative to conventional cementation processes. Also for calcium ions and urea cheap and environmentally compatible alternatives have to be found. While calcium chloride is a by-product of the Solvay synthesis and therefore readily available as a waste stream, urea from Haber–Bosch synthesis is highly energy dependent. The usage of urine as urea source is a promising approach for another step towards an ecological process of MICP. Few studies have already started to investigate the process of MICP using urine from pigs (Chen et al. 2019), cows (Comadran-Casas et al. 2022) and humans (Lambert and Randall 2019). Seawater appears to be a source for free calcium. Although there is only a concentration of about 10 mM calcium in seawater, Cheng et al. (2014) were able to stabilize sand up to an UCS of 300 kPa with seawater as sole calcium source. Yang et al. (2021) used concentrated seawater and were able to consolidate sand columns with UCS of 653 kPa over a duration of 4.5 days. While the low concentrations of calcium ions in seawater might limit the possibility to achieve high compressive strengths over a short duration of time that might be necessary for some applications these studies show the potential that seawater as the sole calcium source can have for MICP especially in marine environment. Another possible source are calcium ions from dissolved eggshells, oysters and scallops. During the process the calcium carbonate from these sources is dissolved in acid and afterwards the pH neutralized. Liang et al. (2020) consolidated poorly graded sand and achieved UCS of 649.7 kPa with dissolved eggshells as sole calcium source. While these approaches are important for the overall development of the process there is still more research necessary concerning the basic mechanisms and influences of the cementation solution composition that can then be transferred towards more environmentally friendly sources of urea and calcium. Besides the environmental impact of MICP there is another challenge in the biocementation itself. Most protocols for biocementation of sand show clogging to some degree during the process due to CaCO_3_ crystals forming close to the injection points. This leads to an inhomogeneous distribution of CaCO_3_ and compressive strength of the samples to a certain degree for all developed protocols (Cheng et al. 2019; Cheng and Cord-Ruwisch 2012; Cheng and Shahin 2016; Wen et al. 2020; Zhao et al. 2014). Especially for the production of novel construction materials this issue needs to be addressed in the future. Standardized production and testing protocols will be necessary to transfer this technology to a larger scale and achieve widespread acceptance and implementation of MICP in the construction industry.

## Concluding remarks and prospects for future work

While there is a lot of knowledge about influencing factors on the results of ureolytic MICP there is still the necessity of understanding these factors on an independent level. This is especially true for the influence of ureolytic activity on the result of MICP. There is sufficient evidence that the rate of ureolysis impacts the morphology and size of CaCO_3_ crystals which again impacts mechanical parameters of treated samples. But this knowledge often derives from studies with varying biomass concentrations, bacterial strains, temperatures and compositions of cementation solution. These parameters impact the rate of ureolysis directly or indirectly. Ureolytic activity correlates directly with the biomass in the system. The specific ureolytic activity varies between ureolytic strains for example due to differences in enzyme expressions for urease or urea transporters. Temperature impacts the rate of ureolysis just like any other enzymatically catalyzed reaction and calcium ions in certain concentrations can inhibit the rate of ureolysis. The separation of these parameters for example by the utilization of GMO´s is certainly a topic for future research that will benefit the development of MICP as a technology. An increase in the usage of GMO´s will also benefit understanding the basic processes during MICP. While it might be sufficient for a lot of experimental studies to monitor the rate of ureolysis through conductivity measurements or colorimetric assays to determine the rate of ammonium production there is still a gap in understanding the separate steps of the ureolysis starting with urea uptake of the cells up to the precipitation kinetics of CaCO_3_ outside of the cell. Increasing the knowledge of these fundamental mechanisms will allow researchers to develop better kinetic models for MICP instead of relying on experimental observations. During the past decades of research concerning ureolytic driven MICP the used strains were almost exclusively cultivated in complex medium. Optimization of cultivation media therefore happened mostly through supplementing complex media with cheap nutrient sources. The publication of a few studies on the cultivation of the most commonly used strain of *Sporosarcina. pasteurii* in defined media in the past years could be the base on which future research could improve the knowledge of the necessary components during cultivation of these strains. While the production of ureolytic microorganisms for MICP in defined medium will most likely not be possible due to the higher cost of these media this will help to move to a more structured approach for media optimization for example through the supplement of complex media with certain vitamins or amino acids. The treatment of ammonium as a toxic by-product is another problem that has to be solved on the adaptation of MICP as a technology. The utilization of ammonium ions through struvite precipitation is a promising technology that gained attention in recent years and deserve a closer look in future studies. Although the usage of zeolites during MICP and the use of low phase injection seem to be able to reduce ammonia leakage during the treatment process significantly ammonium ions will still be leaked into the environment which would not solve the problem long term these technologies might still help to mitigate the ammonia leakage which could make a treatment of ammonium containing waste streams through oxidation easier. Although there are still challenges and problems that need to be solved, MICP is a promising technology with a wide field of possible applications like improvement of existing construction materials, improving mechanical parameters of soils or the production of novel construction material. With increasing knowledge of the fundamental mechanisms and the utilization of cheap and environmentally friendly alternative resources in the coming years MICP can overcome these challenges and get closer to a widespread industrial application of the technology.
